# Anti-inflammatory management for tendon injuries - friends or foes?

**DOI:** 10.1186/1758-2555-1-23

**Published:** 2009-10-13

**Authors:** Kai-Ming Chan, Sai-Chuen Fu

**Affiliations:** 1 Department of Orthopaedics and Traumatology, Faculty of Medicine, The Chinese University of Hong Kong, Hong Kong, PR China

## Abstract

Acute and chronic tendon injuries are very common among athletes and in sedentary population. Most physicians prescribe anti-inflammatory managements to relieve the worst symptoms of swelling and pain, including non-steroidal anti-inflammatory drugs, corticosteroids and physical therapies. However, experimental research shows that pro-inflammatory mediators such as prostaglandins may play important regulatory roles in tendon healing. Noticeably nearly all cases of chronic tendon injuries we treat as specialists have received non-steroidal anti-inflammatory drugs by their physician, suggesting that there might be a potential interaction in some of these cases turning a mild inflammatory tendon injury into chronic tendinopathy in predisposed individuals. We are aware of the fact that non-steroidal anti-inflammatory drugs and corticosteroids may well have a positive effect on the pain control in the clinical situation whilst negatively affect the structural healing. It follows that a comprehensive evaluation of anti-inflammatory management for tendon injuries is needed and any such data would have profound clinical and health economic importance.

## 

Sporting injuries, including both acute trauma and chronic overuse, are usually presented with clinical signs of swelling and pain. Most sport medicine physicians prescribe anti-inflammatory managements to control the pain. However, there are increasing evidences about the unfavorable side effects on the use of various anti-inflammatory agents for tendon injuries.

## Do we need to re-visit the rationale of this practice?

**Figure 1:**
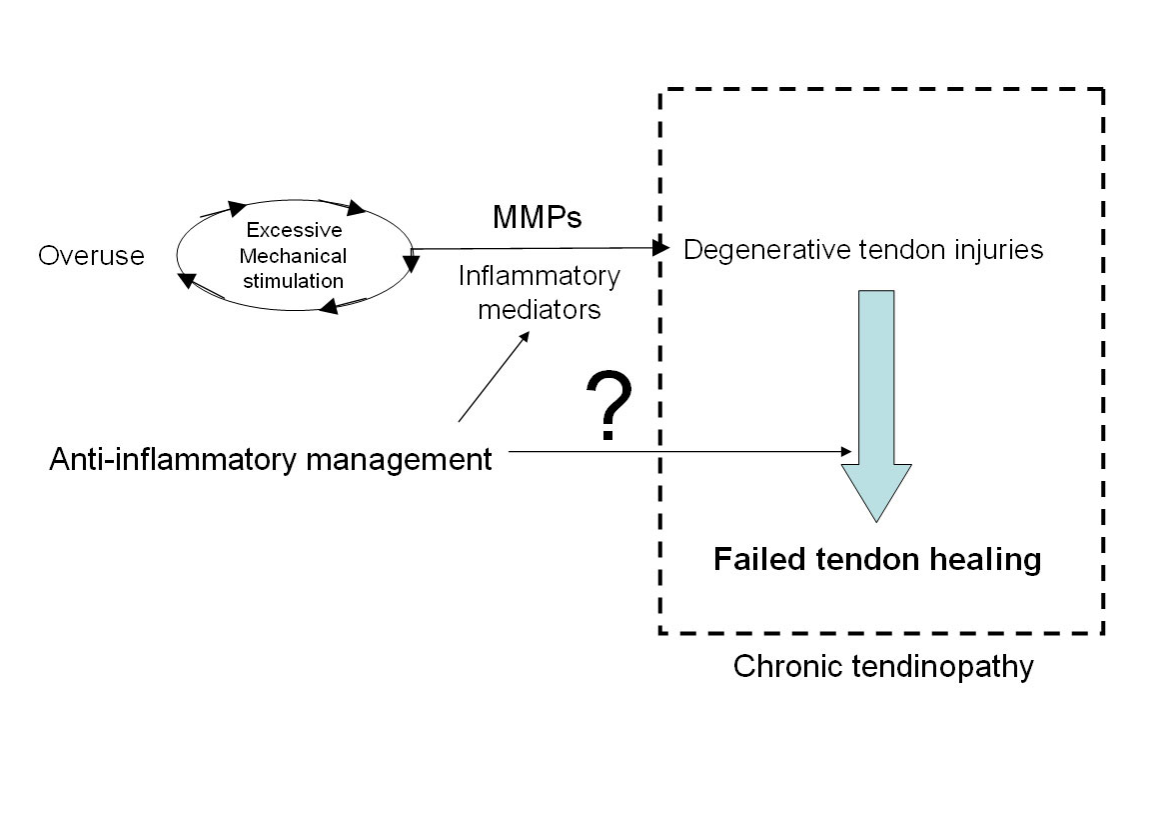
**Overuse or excessive mechanical stimulation to tendons can lead to elevation of metalloproteinases (MMPs) which mediate collagen degradation and hence degenerative tendon injuries**. Healing responses are activated but failed to repair the degenerative injuries, resulting in tendinopathy. It is possible that anti-inflammatory management may affect the development of degenerative injuries as well as the pathological processes of failed healing in tendinopathy.

In cases of chronic tendinopathy, a lack of inflammatory infiltration in the biopsies has challenged the status of "tendinitis". However, it did not rule out the possibility of an inflammatory response in the early development of tendinopathy. Overuse or repetitive stretching on tendons triggered the release of pro-inflammatory mediators, which can induce expression of metalloproteinases and leads to collagen degradation. These findings suggest that inflammatory responses play a key role in the development of degenerative overuse tendon injuries. Together with the fact that all chronic tendinopathy cases received anti-inflammatory management, it is logical to assume that suppression of inflammatory responses may interact with the failed healing of degenerative tendon injuries. We coined the term failed healing to describe the histopathological characteristics of tendinopathy samples, which exhibited traits of both active repair and degenerative injuries. The development of overuse animal model clearly demonstrated the casual relationship of overuse and degenerative tendon injuries through activation of inflammatory mediators [1], but whether the injured tendons failed to heal in these models were not adequately described. On the other hand, animal models of collagenase-induced degenerative injuries revealed failed healing [2] and longstanding pain [3]. We believe that chronic tendinopathy is a result of failed healing of degenerative injuries (Figure 1). The reasons why the degenerative injuries fail to heal might be the core of the mystery. Since pro-inflammatory mediators affect various cellular activities related to tendon healing, it is possible that anti-inflammatory agents might negatively affect tendon healing and contribute to the development of tendinopathy.

## Non-steroidal anti-inflammatory drugs (NSAIDs)

It is still a common practice to prescribe non-steroidal anti-inflammatory drugs (NSAIDs) which primarily work by inhibiting the production of pro-inflammatory prostaglandins. Interestingly, both beneficial and deleterious effects of NSAIDs on tendon healing were reported. It appears that NSAIDs exerted beneficial effects, if any, by influencing the remodeling of collagen matrix, resulting in reduction of cross-sectional area of the healing tendons but tensile strength may or may not be affected. NSAID may also negatively affect early tendon healing, as prostaglandin E_2 _(PGE_2_) is essential for early tendon healing such as control of vascular flow. We doubt that the unchecked use of NSAIDs may exert negative effects when prescribed to tendinopathy patients. As increased expression of cyclooxygenase 2 (COX-2) and increased production of PGE_2 _were found in chronic tendinopathy samples [4], the involvement of PGE_2 _for the development of chronic tendinopathy was implied. As PGE_2 _may mediate the development of overuse tendon injury by induction of metalloproteinases, it is possible that suppression of PGE_2 _may have reduced the extent of degenerative injuries but the normal matrix remodeling would also be affected, which may contribute to the failed tendon healing. Further investigation is urgently needed to disseminate the double-edged properties of prostaglandins in tendon healing before we could advocate the use of NSAIDs for chronic or acute tendon injuries.

## Corticosteroids

Corticosteroids are strong anti-inflammatory agents and peri-tendinous injection of corticosteroid is commonly used to treat the chronic pain in tendinopathy. However, the use of corticosteroid may increase risk of spontaneous ruptures and the deleterious effects of corticosteroid were demonstrated on culture human tendon fibroblasts, including cell viability, proliferation and matrix synthesis [5]. In spite of its potential hazards, corticosteroid injections are still given indiscriminately in many sport clinics!! There is no doubt that the adverse effect of corticosteroids on tendon cells would affect the healing responses to degenerative injuries, corticosteroid injection should be considered as a last resort with careful control on the dosages.

## Other anti-inflammatory modalities

Apart from NSAIDs and corticosteroids, the use of physical therapies to reduce inflammatory signs in tendon injuries is common, but rigorous scientific studies on the efficacy and the underlying mechanisms are not available. Pulsed electromagnetic fields and low level laser treatments may reduce inflammation, probably by suppressing PGE_2 _production. The therapeutic effect of extracorporeal shockwave therapy may be attributed to its anti-inflammatory actions through modulation of nitric oxide production. It appears that these biophysical interventions also exerted anti-inflammatory actions through modulation of pro-inflammatory mediators. As they are relatively safe, further exploration to consolidate their efficacies may yield a better clinical practice for anti-inflammatory management for tendon injuries.

It would be a breakthrough if we could identify disturbed prostaglandin levels as one of the causes of failed healing in tendinopathy, which will certainly guide the development of effective treatment strategies for overuse tendon injuries. In most clinical practice, it is very difficult to monitor the use of anti-inflammatory management for chronic tendinopathy, let alone restricting it in a rigid protocol.
